# Does academic performance or personal growth share a stronger association with learning environment perception?

**DOI:** 10.5116/ijme.57a6.f141

**Published:** 2016-08-28

**Authors:** Jorie M. Colbert-Getz, Sean Tackett, Scott M. Wright, Robert S. Shochet

**Affiliations:** 1 Department of Internal Medicine, University of Utah School of Medicine, USA; 2 Division of General Internal Medicine, Johns Hopkins Bayview Medical Center, Johns Hopkins University School of Medicine, USA

**Keywords:** Learning environment, medical students, personal growth, education environment, academic performance

## Abstract

**Objectives:**

This study was conducted to characterize the relative strength of associations of learning environment perception with academic performance and with personal growth.

**Methods:**

In 2012-2014 second and third year students at Johns Hopkins University School of Medicine completed a learning environment survey and personal growth scale. Hierarchical linear regression analysis was employed to determine if the proportion of variance in learning environment scores accounted for by personal growth was significantly larger than the proportion accounted for by academic performance (course/clerkship grades).

**Results:**

The proportion of variance in learning environment scores accounted for by personal growth was larger than the proportion accounted for by academic performance in year 2 [R^2^Δ of 0.09, F_(1,175)_ = 14.99,  p < .001] and year 3 [R^2^Δ of 0.28, F_(1,169)_ = 76.80, p < .001]. Learning environment scores shared a small amount of variance with academic performance in years 2 and 3.  The amount of variance between learning environment scores and personal growth was small in year 2 and large in year 3.

**Conclusions:**

Since supportive learning environments are essential for medical education, future work must determine if enhancing personal growth prior to and during the clerkship year will increase learning environment perception.

## Introduction

The learning environment in medical school includes the physical space, social interactions, curriculum, and psychological context for students.  How students process and make meaning of their learning environment influences how they develop behaviors and form identities as physicians.^1^^-^^3^ The learning environment has been a focus of research since the 1960s, with new learning environment assessment tools being developed each decade.^4^  Recent research has investigated how factors influence medical students’ perceptions of the learning environment. Specifically, students with a higher learning environment perception perform better on course examinations^5^ and on the United States Medical Licensing Examination (USMLE) Step^1^^,^^6^ spend more time observing physicians or participating in consults during clerkships,^7^ and self-report a higher level of personal growth.^3^^,^^8^  Not surprisingly, burnout among medical students has been shown to be negatively associated with learning environment perception.^9^   Trying to achieve clarity on the practical significance^10^ of these results is difficult because proportions of variance in learning environment scores accounted for by academic performance, time with physicians, personal growth, or burnout scores was not provided in each study.

In considering factors associated with learning environment perceptions it is unknown if the strength of the relationships are the same or different at various time points in medical school. As students progress through the medical school curriculum from a predominant pre-clinical to a predominant clinical learning experience, the learning environment changes in significant ways, as a more structured pedagogy in the medical school building gives way to social learning in the academic medical center filled with new expectations, transitions, uncertainties, stress, and potentially social isolation.^11^^-^^12^  In addition, adapting to learning in clinical settings typically involves processing of interpersonal experiences and relationships, emotions, modeling, and incorporating feedback into future performance.^3^^,^^13^ Thus, as the learning environment changes, the ability to adapt and thrive in clerkships may depend more on personal qualities that permit a student to engage in a relational and reflective fashion, and may depend less on cognitive or academic aspects.  This in turn could influence how students perceive their success in navigating the learning environment, resulting in changes in the way particular factors vary in their strength of association with pre-clinical compared to clinical learning environment perception.

We conducted this study to measure the relative strength of associations of learning environment perception with academic performance and with personal growth at the end of the pre-clinical curriculum (traditional year 2 in United States medical schools) and the end of the core clerkship year (traditionally year 3 in United States medical schools).  Based on prior research^3^^,^^5^^,^^6^^-^^8^ we expected positive relationships between learning environment perception and both academic performance and personal growth.  Additionally, situational cognition theory suggests that outcomes of learning are situated or dependent on the learning environment^14^ and thus academic performance should relate to learning environment perception.   Since prior research has not investigated personal growth and academic performance together we did not know which factor would share a stronger association with learning environment scores, and how they might vary in importance by phase of medical school training.

## Methods

### Study design

This was a cross-sectional study relying on a convenience sample of 178 second year medical students and 172 third year medical students or 350 students total.

### Participants and setting

Between May-June of 2012, 2013, and 2014 second and third year medical students from two consecutive classes (expected graduation year 2014 or 2015) at Johns Hopkins University School of Medicine were invited to complete an online questionnaire.  The questionnaire included a learning environment survey and personal growth scale. Students who completed the questionnaire had a chance to win 1 of 12 $60 gift certificates to a local restaurant.  The Institutional Review Board at Johns Hopkins University School of Medicine approved this study.  All data were stored on a password-protected computer and no identifying information was stored to protect participants’ anonymity. There were no curriculum differences for students in the classes of 2014 and 2015 and both cohorts participated in a learning communities program. 

### Study measures

#### Learning environment perception

Students’ perception of the learning environment was measured with the 28-item Johns Hopkins Learning Environment Survey (JHLES).^3^ Students responded to each 28 item using a 1-5 Likert scale based on their perceptions from day 1 of medical school to the present.  Although Shochet et al.^3^ computed a total score by summing across all survey items in the original citation of JHLES we decided to average item scores for each domain and then sum these scores across the 7 domains resulting in a possible range of 1-35 with higher values indicating more positive endorsement. Summing average domain scores allowed us to treat each domain equally since the number of items varied by domain.  Validity evidence for content, response process, internal structure, and relationship to other variables has been provided in a prior study.^3^

#### Personal growth

Students’ personal growth was measured with a revised personal growth scale.^15^  We selected the top three items from each of the two subscales based on factor analysis loadings, and omitted “humanistic approach to patients” since year 2 students do not see patients as much as year 3 students.  We also split “clarity of goals, values, and direction” into two items. Students indicated the extent to which they were worse or better compared to when they started medical school for each item using a -2 to +2 Likert scale where -2 = much worse, -1 = worse, 0 = no change, +1 = better and +2 = much better.  Summing ratings for the seven items created a personal growth score with a possible range of -14 to 14. Negative scores indicated a decline in growth, positive scores indicated an increase in growth, and a score of zero indicated no change. Validity evidence for this scale has been provided in a sample of residents in the form of content, response process, internal structure and relationship to other variables.^15^

#### Academic performance

An end-of-year 2 academic performance variable was computed by averaging final percent scores (0-100%) in year 1 and year 2 courses.  An end-of-year 3 academic performance variable was computed by averaging numerical scores (Fail = 0, Pass = 1, High Pass = 2, and Honors = 3) for all required clerkships (Internal Medicine, Emergency Medicine, Pediatrics, Psychiatry, Neurology, Surgery, Women’s Health) completed during year 3.

### Data analyses

All data were analyzed with SPSS version 21 (IBM Corp., Armonk, New York). Descriptive statistics were calculated for demographic variables, JHLES scores, personal growth scores, and academic performance.  To assess whether survey responders were representative of all medical students, distributions of gender and race were compared between survey responders and all students with Mann Whitney U tests for years 2 and 3 separately. JHLES scores and personal growth scores were also compared between year 2 and year 3 students with Mann-Whitney U tests.

Separate hierarchical linear regression analyses were employed for year 2 and year 3 to determine if the proportion of variance in learning environment scores accounted for by personal growth was significantly larger than the proportion accounted for by academic performance.  To perform hierarchical linear regression analysis, academic performance and personal growth were entered in two models.  In model 1, JHLES scores were regressed on academic performance.  In model 2, JHLES scores were regressed on both personal growth scores and academic performance and the two models were statistically compared to each other with a R^2^Δ value. The delta value provides a measure of the difference between the R^2^ value in model 1 and the R^2^ value in model 2. A significant R^2^Δ value would indicate personal growth scores accounted for a significantly larger proportion of variance in JHLES scores than the variance accounted for by academic performance.  Percentages of variance were computed between JHLES scores for both academic performance and personal growth with squared correlation coefficients, and for each variable uniquely with squared partial correlation coefficients.  A value above 24% indicates a high degree of variance, a value between 9%-24% indicates a moderate degree of variance, and a value less than 9% indicates a small degree of variance between two variables.^16^

To investigate associations of JHLES scores, academic performance, and personal growth scores in years 2 and 3, we identified a subset of students who started year 3 immediately after year 2, and completed all survey items in both years.  At Johns Hopkins School of Medicine, students may take time off between years 2 and 3 for leave of absence, research, and other degree programs.  To ensure the subset was not different from the overall sample, we verified the results of the hierarchical linear regression analyses.  We then computed correlations between JHLES, personal growth, and academic performance scores in years 2 and 3 with Spearman’s rho.  Finally, students were grouped into one of four categories based on their personal growth scores in years 2 and 3: (1) positive personal growth scores in both years, (2) positive personal growth score in year 2 only, (3) positive personal growth score in year 3 only, or (4) negative personal growth score and/or a score of zero (no change) in both years. Year 3 JHLES scores were compared among the four groups with ANOVA and Schefflé post-hoc tests were conducted as appropriate.

## Results

Response rates were 81% (178) for second year students and 72% (172) for third year students. Table 1 provides demographic information for respondents.  Distributions of gender and race were not significantly different between respondents and all medical students in year 2 (p> 0.49 for gender and race) or year 3 (p> 0.45 for gender and race) suggesting that the sample was representative of all medical students at the Johns Hopkins University School of Medicine.  Table 1 also provides means for JHLES scores, personal growth scores, and academic performance for survey respondents. JHLES and personal growth scores were not significantly different between year 2 and year 3 students (p> 0.75).

**Table 1:** Demographics and mean JHLES scores, personal growth score, and academic performance for 178 year 2 and 172 year 3 medical students

Student characteristic	Year 2 N (%)		Year 3 N (%)	
Gender				
Female	94 (53)		82 (48)	
Male	84 (47)		90 (52)	
Race				
Asian	55 (31)		57 (33)	
Caucasian	96 (54)		91 (53)	
Underrepresented minority^*^	25 (14)		23 (13)	
Unknown	2 (1)		1 (1)	
Study Measures	Mean(Standard Deviation)		Mean(Standard Deviation)	
JHLES^†^	26.98 (3.22)		27.07 (3.38)	
Personal Growth^‡^	2.72 (3.95)		2.65 (4.77)	
Academic Performance^¶^	87% (4%)		2.25 (0.49)	

^*^Includes all students who self-identified as Pacific Islander, American Indian, African American or Hispanic (alone or in combination with another racial category).

^†^Johns Hopkins Learning Environment Survey (JHLES) scores have a possible range of 1-35 with higher values reflecting more positive endorsement of the learning environment.

^‡^Personal Growth scores have a range of -14 to +14. Negative scores indicate a decline in growth, positive scores indicate an increase in growth, and a score of zero indicates no change.

^¶^Academic performance was measured on a 0-100% scale for year 1-2 courses and 0(fail), 1(pass), 2(high pass), 3(honors) scale for required clerkships in year 3.

Hierarchical linear regression analyses showed that for year 2 students, academic performance was not significantly related to JHLES scores in model 1, R^2^= 0.02, F_(1,176)_ = 2.40, β=0.12, p= 0.12. In model 2, academic performance and personal growth scores were significantly related to JHLES scores (R^2^= 0.08), and personal growth scores accounted for a significantly larger proportion of variance in JHLES scores than academic performance, R^2^Δ of 0.06, F_(__1,175)_ = 14.99, β=0.25, β=0.12,  p= 0.01. For year 3 students, academic performance was significantly related to JHLES scores in model 1, R^2^ = 0.08, F_(__1,170)_ = 14.39, β=0.28, p< 0.001.   In model 2, academic performance and personal growth scores were significantly related to JHLES scores (R^2^ =0.38), and personal growth scores accounted for a significantly larger proportion of variance in JHLES scores than academic performance, R^2^Δ of 0.30, F_(__1,169)_ = 80.22, β=0.16, β=0.56, p< 0.001.  In year 3, personal growth scores shared a large amount of variance with JHLES scores (30%) while the amount of variance between these two variables was small in year 2 (6%). Additionally, academic performance shared a small amount of variance with JHLES scores for both years 2 (4%) and 3 (2%).

There were 129 students who started year 3 immediately after year 2 and completed all survey items in both of those years.  Hierarchical linear regression analyses for these students were similar to the entire sample, with personal growth scores accounting for a significantly larger proportion of variance in JHLES scores than academic performance for year 2, R^2^Δ = 0.04, p= 0.02, and year 3, R^2^Δ = 0.26, p< 0.001. There were strong positive correlations between year 2 and year 3 JHLES scores, (Spearman’s rho = 0.69), year 2 and year 3 personal growth scores, (Spearman’s rho = 0.54), and year 2 and year 3 academic performance (Spearman’s rho = 0.50).

The majority of these 129 students (52%, 67) had positive personal growth scores in years 2 and 3. Sixteen percent (20) had a positive personal growth score in year 2 only, and 12% (16) had a positive personal growth score in year 3 only.  Twenty percent (26) had negative personal growth scores and/or scores of zero in years 2 and 3. 

Year 3 JHLES scores significantly varied among the four groups, F_(__3,125)_ = 9.21, p < 0.001, $\eta_{p}^{2}$ = 0.18.  Post-hoc analysis showed this effect was due to higher JHLES scores for students who experienced positive personal growth in year 3 only (mean JHLES score = 28) or positive personal growth in years 2 and 3 (mean JHLES score = 28) compared to students who had negative or no growth in years 2 and 3 (mean JHLES score = 24), p= 0.02 and p< 0.001, respectively. All other post-hoc analyses were not significant, p> 0.11. 

## Discussion

Personal growth scores accounted for a significantly larger proportion of variance in learning environment scores than did academic performance.  From our data, it appears as if academic performance may only be minimally related to learning environment perception as assessed by the JHLES. This finding surprised us because our a priori hypotheses was that people performing well academically would believe that the learning environment was working for them, and those not performing at the highest levels would be less satisfied with the learning environment. Interestingly, the amount of variance between personal growth and learning environment perception was greater at the end of the clerkship year than it was at the end of the pre-clinical phase in this study.  Students with positive personal growth scores at the end of the clerkship year and/or at the end of pre-clinical phase rated the learning environment more favorably than did students who has negative personal growth scores and/or no change in growth (a score of zero) in years 2 and 3.

Prior studies have found a positive relationship between academic performance and learning environment perception as measured by the Dundee Ready Educational Environment Measure (DREEM)^7^ and the Learning Environment Questionnaire (LEQ).^6^  One reason for the minimal relationship between academic performance and learning environment perception in this study could be that we used a new tool (JHLES) that measures seven domains of the learning environment, including the social and relational process.  Each domain was weighted equally for computing JHLES scores.   The DREEM and the LEQ were developed prior to 2000 and proposed to capture 3-5 domains. If academic climate was one learning environment domain out of 3-5 domains compared to one out of 7 domains the relationship between overall learning environment perception and academic performance would be less pronounced for the 7 domain tool compared to the 3-5 domain tool.

We did find the proportion of variance in learning environment scores accounted for by personal growth and accounted for by academic performance to vary by training phase. The early pre-clinical phase of training in medical school is certainly a more familiar experience for those transiting from college to medical school given that it is has a highly structured curricula and is intentionally designed for medical students to build a foundation.  Thus, learning in the pre-clinical phase of medical school is focused on pedagogy with unambiguous curricular goals and structured and predictable assessments.  In the clinical years, the environment is more complex, constantly changing, and involves social awareness and engagement for learning, rather than primary reliance on structured pedagogy.  Further, clerkship rotations require that the student take a more active role in their learning. On top of that, the need of another, “the patient”, supersedes all.  Some students struggle with this shift to being a self-directed learner.^12^ The structured pre-clinical years may allow some students to have a favorable perception of the learning environment even if they have not realized substantive personal growth.  By the end of the clerkship year, personal growth and learning environment perception share a stronger association than in year 2, such that positive growth may be necessary for students to appreciate or derive meaning from the clinical learning environment.

### Limitations

Several limitations of this study should be considered. First, we used a cross sectional, rather than longitudinal design for the main analyses.  We decided to do this because we did not want to limit the results to only those students who start year 3 immediately after year 2.  This analytic plan allowed us to capture more students and therefore have higher power for the cross sectional design; the results were verified in the subset sample. Second, this study was conducted at one institution. Since learning environments vary by institution, the strength of associations between personal growth and learning environment perception may be different at other medical schools. Finally, our research design only allowed us to make assertions about associations between variables, not if one variable predicted the other variable.

### Implications and recommendations for future research

All medical schools are being asked to thoughtfully consider and measure the learning environment.  The analyses in this manuscript involved multiple points in time during the medical school experience and explored the practical significance of results by quantifying how much variance was accounted for in learning environment perception by personal growth scores and by academic performance.  Both analytic approaches make novel contributions to the learning environment literature. Future research needs to investigate if lower perceptions of a learning environment can be improved by enhancing students’ personal growth prior to and during the clerkship years.

### Acknowledgements

The authors wish to thank the Student Outcomes Research Database (SORD) Advisory Board for assistance in accessing medical students’ academic performance data.

### Conflict of Interest

The authors declare that they have no conflict of interest.

## References

[1] Branch WT (2000). Supporting the moral development of medical students.. J Gen Intern Med.

[2] Suchman AL, Williamson PR, Litzelman DK, Frankel RM, Mossbarger DL, Inui TS (2004). Toward an informal curriculum that teaches professionalism. Transforming the social environment of a medical school.. J Gen Intern Med.

[3] Shochet RB, Colbert-Getz JM, Wright SM (2015). The Johns Hopkins learning environment scale: measuring medical students' perceptions of the processes supporting professional formation.. Acad Med.

[4] Colbert-Getz JM, Kim S, Goode VH, Shochet RB, Wright SM (2014). Assessing medical students' and residents' perceptions of the learning environment: exploring validity evidence for the interpretation of scores from existing tools.. Acad Med.

[5] Mayya S, Roff S (2004). Students' perceptions of educational environment: a comparison of academic achievers and under-achievers at kasturba medical college, India.. Educ Health (Abingdon).

[6] Wayne SJ, Fortner SA, Kitzes JA, Timm C, Kalishman S (2013). Cause or effect? The relationship between student perception of the medical school learning environment and academic performance on USMLE Step 1.. Med Teach.

[7] van Hell EA, Kuks JB, Cohen-Schotanus J (2009). Time spent on clerkship activities by students in relation to their perceptions of learning environment quality.. Med Educ.

[8] Tackett S, Bakar HA, Shilkofski NA, Coady N, Rampal K, Wright S (2015). Profiling medical school learning environments in Malaysia: a validation study of the Johns Hopkins Learning Environment Scale.. J Educ Eval Health Prof.

[9] Dyrbye LN, Thomas MR, Harper W, Massie FS, Power DV, Eacker A, Szydlo DW, Novotny PJ, Sloan JA, Shanafelt TD (2009). The learning environment and medical student burnout: a multicentre study.. Med Educ.

[10] Regehr G (2001). Reporting of Statistical Analyses.. Academic Medicine.

[11] Teunissen PW, Westerman M (2011). Opportunity or threat: the ambiguity of the consequences of transitions in medical education.. Med Educ.

[12] Krupat E, Pelletier SR, Chernicky DW (2011). The third year in the first person: medical students report on their principal clinical year.. Acad Med.

[13] Hirsh DA, Ogur B, Thibault GE, Cox M (2007). "Continuity" as an organizing principle for clinical education reform.. N Engl J Med.

[14] Durning SJ, Artino AR (2011). Situativity theory: a perspective on how participants and the environment can interact: AMEE Guide no. 52.. Med Teach.

[15] Wright SM, Levine RB, Beasley B, Haidet P, Gress TW, Caccamese S, Brady D, Marwaha A, Kern DE (2006). Personal growth and its correlates during residency training.. Med Educ.

[16] Cohen J (1992). A power primer.. Psychological Bulletin.

